# Oral care with human milk is associated with increased human milk feeding and breastfeeding for newborns with critical congenital heart disease

**DOI:** 10.1038/s41372-026-02698-7

**Published:** 2026-04-27

**Authors:** Kristin M. Elgersma, Nancy L. Slater, Kimberly Watkins, Lynn R. Tanner, Nellie M. Swanson, Sara E. Ramel

**Affiliations:** 1 School of Nursing, University of Minnesota, Minneapolis, MN USA; 2 Children’s Minnesota, Minneapolis, MN USA; 3 The Children’s Heart Clinic, Minneapolis, MN USA; 4 Department of Pediatrics, Medical School, Minneapolis, MN USA

**Keywords:** Congenital heart defects, Paediatrics

## Abstract

**Objective:**

Describe the prevalence of oral care with human milk (OHM) for infants with critical congenital heart disease (CCHD) and estimate the effect of early OHM (1st postnatal week) on lactation outcomes.

**Study design:**

Retrospective cohort including infants with CCHD from 2014–2023. Adjusted regression estimated effects of early OHM frequency (quartiles (Q); Q1 = 0–1 OHM doses, Q2 = 2–6, Q3 = 7–13, Q4 ≥ 14) on human milk intake and breastfeeding (BF) at discharge.

**Result:**

For 297 infants ≤6 months old, OHM comprised 25.5% of oral care. Early OHM frequency was associated with 42.67–57.03 mL/kg/d higher human milk intake (*p* < 0.001 for Q2–4 compared to Q1), and 4.7–6.03 greater odds of BF (*p* < 0.01 for Q2–4) at discharge.

**Conclusion:**

Increased early OHM frequency was strongly associated with lactation outcomes for newborns with CCHD. OHM may support human milk/BF exposure in this vulnerable population.

## Introduction

Oral care with human milk (OHM) is a standard neonatal intensive care unit (NICU) intervention for infants who are not able to feed orally. The most structured version of this practice, known as oral immunotherapy, involves placing a small amount (eg, 0.2 mL) of maternal human milk on the inside of the infant’s cheek via syringe or swab, and is typically performed every 3–6 h in place of expected oral feeding [1]. More broadly, OHM may include general oral care using maternal human milk in place of another substance such as mouthwash or sterile water [2, 3]. OHM is typically initiated shortly after birth and is intended to provide an infant with exposure to colostrum, which is rich in bioactive components that support passive immunity transfer, offer protection from pathogens, and support healthy gut microbiome colonization [4, 5].

Several recent systematic reviews and meta-analyses of randomized controlled trials have summarized the evidence on clinical benefits of OHM for preterm and low birth weight populations, finding reductions in necrotizing enterocolitis [6–10], sepsis [6, 7, 9, 10], ventilator-associated pneumonia [8–10], duration of mechanical ventilation [8], feeding intolerance [6], time to full feeding volume [6, 7, 9–11], length of hospital stay [8, 9, 11], and mortality [6, 10, 12]. Complementary mechanistic studies suggest that these clinical improvements may be mediated by immunomodulatory effects of OHM, including increased levels of immune-protective factors such as secretory IgA [13–16], IgM [15], lactoferrin [14–16], and IL-1ra [17], and decreased proinflammatory cytokines and chemokines (eg, IL-1β, Il-6, IL-8, TNF-α, IFN-γ)[14, 17].

OHM has also been recommended as an intervention to promote lactation and facilitate the transition to direct breastfeeding (BF) in the NICU [18–20]. One study suggested that OHM may increase human milk feeding for very low birth weight infants at 6 weeks and at discharge [21]. Similarly, a study including both preterm and sick term infants (*n* = 3 with cardiac anomalies) identified early colostrum feeding as a predictor of direct BF at NICU discharge [22]. However, the evidence on OHM for infants with critical congenital heart disease (CCHD) is limited [23]. Investigating OHM in infants with CCHD presents challenges, and it is unclear whether the benefits observed in preterm infants are similar in magnitude for infants with CCHD. The clinical trajectory of these infants is highly variable, and the prevalence of OHM in this population is not well characterized. Some infants may experience prolonged periods of nil per os (NPO) with multiple OHM opportunities, while others may have minimal oral feeding restrictions, limiting the need for OHM. We aimed to address knowledge gaps about OHM in CCHD by (1) describing the prevalence and patterns of OHM for infants undergoing neonatal cardiac surgery for CCHD over 10 years at a single site, and (2) estimating associations between early OHM during the first 7 postnatal days and lactation outcomes at discharge.

## Materials/subjects and methods

This retrospective cohort study included infants with CCHD who underwent cardiac surgery with cardiopulmonary bypass ≤30 days old from 2014–2023 at Children’s Minnesota. We included infants ≥36 weeks gestational age who were admitted by day of life 1. Infants were excluded if they were discharged home before neonatal surgery or discharged >6 months old, as guidelines recommend exclusive human milk for infants through age 6 months [24, 25]. Infants who were opted out of research in the electronic health record (EHR) were excluded, and infants who died were excluded from analyses of discharge human milk/BF outcomes. The Children’s Minnesota Institutional Review Board approved this study and deemed it exempt.

### Setting

During the 10-year study period, human milk and BF were supported institutionally through hospital-grade breast pumps for all pumping parents, onsite milk storage, a donor milk program available to all units admitting newborns, meal vouchers for lactating parents, patient education materials, a lactation website, and a “Mother’s Support Group” facilitated by a lactation consultant. Infants with CCHD were cared for in the cardiovascular intensive care unit (CVICU) from birth through discharge. All CVICU newborn admissions received standing orders for feeding therapy consultation, and the primary CVICU feeding therapist held additional certification as a lactation counselor. Lactation consultants were available and reviewed the census on a weekly basis. One barrier to lactation support was initial inadequate staffing to accommodate multiple neonatal units, with 1.2 full-time equivalent (FTE) lactation consultants gradually increasing to 3.2 FTE by the end of the study period.

Site protocols required oral care to be provided every 4 h when an infant was intubated, and twice per day when an infant was extubated but NPO. Several substances were available for oral care, including maternal human milk, mouthwash, water, and commercial formula. Donor human milk was available for enteral feedings, but was not typically used for oral care. During the study period, provision of OHM was not protocolized, but was increasingly encouraged by unit leaders. However, OHM was never formally added to unit order sets, and bedside staff were ultimately responsible for choosing the substance to be used for oral care throughout the 10-year study period.

### Data

All data were extracted from the medical record by site informaticians and through manual chart review. Due to patient privacy regulations and institutional data-use restrictions, the data for this study cannot be made publicly available and cannot be shared.

### Exposures and outcomes

The exposure of interest was early OHM frequency during the first 7 postnatal days. The rationale for selecting this timeframe was two-fold. First, the optimal duration of OHM has not been clearly established. In clinical trials of oral immunotherapy with human milk, implementation ranges from 48 h [26] to the point at which full oral feeding is achieved [27], with all studies initiating immunotherapy as early as possible after birth. Second, the median age at surgery was 5 days, making the first 7 postnatal days a relevant window to capture perioperative OHM opportunities while maintaining consistency across the cohort. We divided the cohort into quartiles as follows: quartile 1 (Q1) received 0–1 OHM doses, quartile 2 (Q2) received 2–6 doses, quartile 3 (Q3) received 7–13 doses, and quartile 4 (Q4) received ≥14 doses. While infants could have theoretically received up to 42 OHM doses in the first postnatal week, not all infants were intubated and/or NPO during this time. Thus, the potential maximum number of doses varied by individual.

The outcomes of interest were the infant’s human milk consumption (mL/kg/day) [28] and any BF [29] (defined as human milk directly from the breast) at discharge. To fully capture infant feeding patterns, “at discharge” was defined as during the last week of hospitalization, since parents of hospitalized infants may be encouraged to use bottles or test commercial formulas before leaving the hospital [30, 31]. As in previous literature [32–34], the focus was the infant’s primary diet, not including fortification or additives. Human milk could include maternal or donor human milk; however, donor human milk use at the time of discharge was rare. Direct BF volume was mostly unmeasured; thus, we estimated the volume of each BF session during the 7 days before discharge to be 35 mL. This was the mean volume of all measured feeding sessions during this time (35.23 mL) and is similar to an average 36 mL per BF session reported for term hospitalized infants [35].

### Analysis

Descriptive statistics and data visualization determined the prevalence and patterns of OHM usage. We initially compared sample characteristics among OHM quartiles using Chi-squared or Fisher’s exact tests for categorical data, and Kruskal-Wallis rank sum tests for continuous variables. To estimate associations between OHM quartiles and lactation outcomes at discharge, we fit unadjusted and adjusted linear or logistic regression models for outcomes of interest, with Q1 as the reference group to which Q2–4 were compared. Model 1 for each outcome included infants ≤6 months old at discharge; Model 2 included infants ≤6 months old who received no preoperative enteral nutrition (which allows focused examination of a group that may be particularly at risk for later oral feeding and lactation challenges) [36, 37]; and Model 3 included infants ≤6 months old whose birthing parent had an EHR-documented human milk goal (for human milk outcomes) or BF goal (for any BF). The parent’s feeding goals were documented at the infant’s ICU admission as part of standard nursing workflow. When this information was not recorded, we reviewed EHR notes from feeding therapists and lactation consultants, who typically discussed the parent’s feeding intentions during their initial consultation.

All models were adjusted for covariates known to be associated with lactation outcomes and available in the EHR: hospital length of stay, insurance type, and parent-reported infant race [37]. Models 1 and 3 were also adjusted for any BF in the first 7 days. Models for human milk mL/kg/d were adjusted for age at surgery, and models for BF at discharge were adjusted for diagnosed vocal cord dysfunction. Additional covariates that were considered but did not improve the models included birth year, single ventricle physiology, and major genetic syndrome. Model diagnostics for linear models included Q-Q plots, residuals versus fitteds plots, and Breusch–Pagan tests. We assessed multicollinearity in binomial models by calculating the variance inflation factor (VIF) for each covariate, and used Akaike Information Criterion scores in all models to inform covariate selection. Robust standard errors were calculated for linear models for conservative estimates, and statistical significance was set at *p* < 0.05.

## Results

**Fig. 1:**
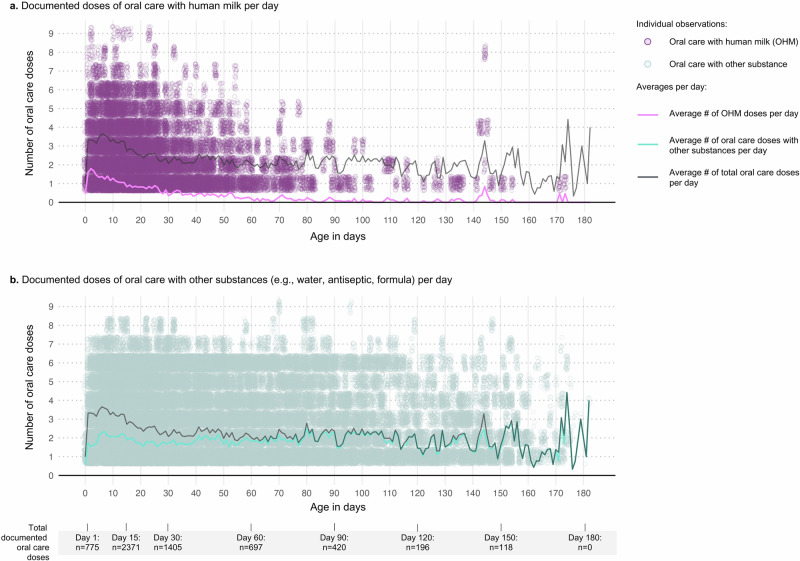
Documented oral care over the first 6 months. **a** Documented doses of oral care with human milk per day. **b** Documented doses of oral care with other substances (e.g., water, antiseptic, formula) per day.

**Fig. 2:**
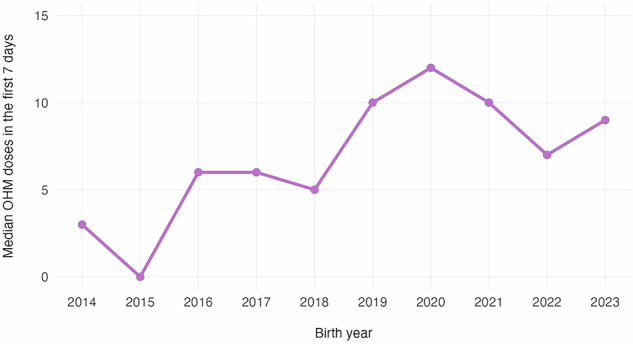
Median oral care with human milk (OHM) doses in the first 7 postnatal days, over time.

Infants (*n* = 297) were 37.4% female, 67.1% white, 38.4% with public insurance, 37.0% with single ventricle physiology, and 11.1% with a major genetic syndrome (Table 1). Throughout the hospital stay, most infants (93.3%) received at least one dose of OHM, and OHM comprised 25.5% of all instances of oral care for infants ≤6 months old (8285/32,506 total doses). In the first 7 days, 82.8% of infants received at least 1 OHM dose with a median 6 (range, 0–31) doses. Figure 1 depicts the frequency of OHM and oral care with other substances (eg, formula, water, mouthwash) during the neonatal surgical hospitalization. This figure illustrates that OHM was used most commonly during the early postnatal period, with an average of less than 1 dose per infant per day by 14 days of age. Oral care with other substances was used most frequently during the remainder of the hospital stay, while OHM use became rare. OHM prevalence in the first week increased over time (Fig. 2), from a median 3 doses per infant in 2014 to 9 doses in 2023, with a high of 12 doses in 2020.

**Table 1:** Sample characteristics, compared by oral care with human milk frequency in the first 7 postnatal days.

	Full sampleᵃ	By quartiles of OHM frequency in the first 7 postnatal days				
	(*n* = 297)	Q1 (0-1 doses) *n* = 68	Q2 (2-6 doses) *n* = 81	Q3 (7-13 doses) *n* = 85	Q4 ( ≥ 14 doses) *n* = 63	*p* valueᵇ
Sex						0.784
Female	111 (37.4%)	29 (42.6%)	29 (35.8%)	30 (35.3%)	23 (36.5%)	
Male	186 (62.6%)	39 (57.4%)	52 (64.2%)	55 (64.7%)	40 (63.5%)	
Race						0.004
Another race/multi-race	32 (11.1%)	10 (14.9%)	10 (12.5%)	8 (9.8%)	4 (6.7%)	
Asian	17 (5.9%)	8 (11.9%)	3 (3.8%)	3 (3.7%)	3 (5.0%)	
Black/African American	46 (15.9%)	17 (25.4%)	11 (13.8%)	12 (14.6%)	6 (10.0%)	
White	194 (67.1%)	32 (47.8%)	56 (70.0%)	59 (72.0%)	47 (78.3%)	
(Unknown)	8	1	1	3	3	
Insurance type						<0.001
Public	114 (38.4%)	39 (57.4%)	33 (40.7%)	24 (28.2%)	18 (28.6%)	
Private/self	183 (61.6%)	29 (42.6%)	48 (59.3%)	61 (71.8%)	45 (71.4%)	
Birth weight-for-age z-score	-0.12 (1.09)	-0.07 (1.04)	0.05 (1.06)	-0.30 (1.15)	-0.14 (1.10)	0.356
Primary cardiac diagnosis						0.448
Coarctation of the aorta/IAA/aortic anomaly	37 (12.5%)	8 (11.8%)	7 (8.6%)	12 (14.1%)	10 (15.9%)	
DORV: Sub-aortic VSD, pulmonary outflow obstruction	14 (4.7%)	1 (1.5%)	6 (7.4%)	4 (4.7%)	3 (4.8%)	
DORV: Sub-pulmonary VSD, aortic/arch obstruction	9 (3.0%)	2 (2.9%)	3 (3.7%)	3 (3.5%)	1 (1.6%)	
HLHS	72 (24.2%)	17 (25.0%)	22 (27.2%)	21 (24.7%)	12 (19.0%)	
Other SV	32 (10.8%)	8 (11.8%)	7 (8.6%)	10 (11.8%)	7 (11.1%)	
TAPVR	15 (5.1%)	6 (8.8%)	3 (3.7%)	4 (4.7%)	2 (3.2%)	
TGA	75 (25.3%)	14 (20.6%)	22 (27.2%)	18 (21.2%)	21 (33.3%)	
ToF	15 (5.1%)	3 (4.4%)	4 (4.9%)	6 (7.1%)	2 (3.2%)	
Truncus arteriosus	12 (4.0%)	0 (0.0%)	4 (4.9%)	5 (5.9%)	3 (4.8%)	
Other	16 (5.4%)	9 (13.2%)	3 (3.7%)	2 (2.4%)	2 (3.2%)	
Single ventricle physiology^c^	111 (37.4%)	27 (39.7%)	30 (37.0%)	33 (38.8%)	21 (33.3%)	0.878
Major genetic syndrome^d^	33 (11.1%)	11 (16.2%)	4 (4.9%)	12 (14.1%)	6 (9.5%)	0.120
Any preoperative feeding	179 (60.3%)	36 (52.9%)	52 (64.2%)	52 (61.2%)	39 (61.9%)	0.543
Any preoperative BF	77 (25.9%)	4 (5.9%)	27 (33.3%)	23 (27.1%)	23 (36.5%)	<0.001
Any preoperative human milk feeding^e^	160 (53.9%)	23 (33.8%)	46 (56.8%)	52 (61.2%)	39 (61.9%)	0.002
# of days on prostaglandin E1	6 (5)	6 (3)	5 (3)	6 (6)	7 (6)	0.327
Age at surgery (days)	5 (3, 8)	4 (2, 8)	4 (3, 6)	5 (3, 9)	6 (3, 11)	0.149
ECMO	54 (18.2%)	19 (27.9%)	10 (12.3%)	14 (16.5%)	11 (17.5%)	0.094
Necrotizing enterocolitis	15 (5.1%)	4 (5.9%)	3 (3.7%)	5 (5.9%)	3 (4.8%)	0.936
Chylothorax	21 (7.1%)	4 (5.9%)	6 (7.4%)	6 (7.1%)	5 (7.9%)	0.979
Vocal cord dysfunction	21 (7.1%)	7 (10.3%)	3 (3.7%)	6 (7.1%)	5 (7.9%)	0.450
# of additional cardiac surgeries this stay	0 (0, 1)	0 (0, 1)	0 (0, 0)	0 (0, 0)	0 (0, 1)	0.068
Neonatal surgical length of stay (days)	31 (21, 54)	35 (22, 68)	30 (19, 48)	35 (21, 53)	31 (22, 56)	0.474
Discharge weight-for-age z-score	-1.33 (1.16)	-1.26 (1.05)	-1.24 (1.19)	-1.52 (1.21)	-1.24 (1.14)	0.384
Discharge length-for-age z-score	-1.28 (1.54)	-1.18 (1.57)	-1.36 (1.40)	-1.43 (1.71)	-1.10 (1.44)	0.819
Deceased before discharge	35 (11.8%)	12 (17.6%)	7 (8.6%)	6 (7.1%)	10 (15.9%)	0.119
Any documented maternal goal to provide human milk	268 (90.5%)	46 (68.7%)	78 (96.3%)	82 (96.5%)	62 (98.4%)	<0.001
Any documented maternal goal to breastfeed	234 (82.4%)	37 (58.7%)	72 (90.0%)	71 (86.6%)	54 (91.5%)	<0.001
Total feeding volume at discharge (mL/kg/d)	139 (30)	132 (30)	140 (25)	139 (32)	146 (34)	0.040
Human milk volume at discharge (mL/kg/d)	92 (68)	46 (61)	101 (61)	103 (63)	110 (71)	<0.001
≥75% human milk at discharge	155 (59.2%)	16 (28.6%)	51 (68.9%)	54 (68.4%)	34 (64.2%)	<0.001
Any BF at discharge	99 (37.8%)	8 (14.3%)	32 (43.2%)	35 (44.3%)	24 (45.3%)	<0.001
Any OHM in the first 7 days	246 (82.8%)	–	–	–	–	
# of OHM doses in the first 7 days	6 (2, 12)	0 (0, 1)	4 (3, 6)	10 (8, 11)	17 (16, 21)	<0.001
Any OHM during the hospitalization	277 (93.3%)	–	–	–	–	
# of OHM doses during the hospitalization	23 (8, 46)	3 (0, 7)	16 (8, 32)	35 (22, 51)	48 (28, 67)	<0.001

^a^ Data are presented as n (%) for categorical variables; mean (SD) for continuous variables, except age at admission, age at surgery, and hospital length of stay are presented as median (25%, 75%).

^b^ *p* values calculated by Chi-squared or Fischer’s exact tests for categorical variables and Kruskal-Wallis rank sum tests for continuous variables.

^c^ Infants were considered to have single ventricle physiology if they (1) had a clear single ventricle diagnosis (e.g., HLHS), (2) had a Norwood surgery as the stage 1 palliation, or (3) progressed to Glenn surgery after initial intervention.

^d^ Major genetic syndromes included genetic and chromosomal anomalies known to have clinically significant sequelae beyond the CHD diagnosis. Examples are 22q11 deletion syndrome, 8p23.1 microdeletion syndrome, Trisomy 8, Trisomy 13, Trisomy 18, Trisomy 21, Monosomy X, Alagille syndrome, VACTERL syndrome, and other clinically significant chromosomal or genetic abnormalities.

^e^ “Human milk” refers to maternal or donor human milk. Donor human milk was not widely used beyond the early neonatal period.

*AVSD* atrioventricular septal defect, *BF* breastfeeding, *DORV* double outlet right ventricle, *ECMO* extracorporeal membrane oxygenation, *HLHS* hypoplastic left heart syndrome, *IAA* interrupted aortic arch, *OHM* oral care with human milk, *SV* single ventricle, *TAPVR* total anomalous pulmonary venous return, *TGA* transposition of the great arteries, *ToF* tetralogy of Fallot.

Associations between OHM frequency in the first 7 days and lactation outcomes at discharge can be found in Table 2. In adjusted analysis, greater OHM frequency was associated with 42.67–57.03 mL/kg/d higher human milk volume at discharge (Table 2a, *p* < 0.001 for Q2, Q3, and Q4 compared to Q1; full sample). These results were consistent for infants with no preoperative feeding (48.09–66.43 mL/kg/d greater human milk, all *p* < 0.05). Limiting the sample to infants with a documented maternal goal to provide human milk, greater OHM frequency was associated with 16.09–29.69 mL/kg/d greater human milk (*p* < 0.05 for Q3 and Q4). Although a portion of this volume was estimated due to unmeasured BF occurrences, only 37.8% of the sample was BF at discharge, and these infants received an average of ~1 BF session per day. In sensitivity analysis excluding all estimated BF volumes, the results were consistent: greater OHM frequency was associated with 37.92–51.73 mL/kg/d higher human milk volume in the full sample (*p* ≤ 0.001 for all quartiles).

**Table 2:** Associations between oral care with human milk frequency in the first 7 postnatal days and human milk or breastfeeding outcomes at discharge.

a. Outcome: Average infant human milk mL/kg/d intake at discharge				
	Estimate (β)	SE	95% CI	*p* value
Model 1: ≤ 6 months old at discharge (*n*=256)				
OHM frequency:^c^				
Quartile 2	42.67	11.57	(33.16, 77.59)	**<0.001**
Quartile 3	49.38	10.79	(34.66, 78.48)	**<0.001**
Quartile 4	57.03	12.40	(39.95, 88.03)	**<0.001**
Model 2: ≤ 6 months old + no preoperative enteral feeding (*n*=98)				
OHM frequency:				
Quartile 2	48.09	20.16	(14.67, 81.50)	**0.017**
Quartile 3	61.49	18.98	(28.39, 94.60)	**0.001**
Quartile 4	66.43	24.61	(28.42, 104.45)	**0.007**
Model 3: ≤ 6 months old + any human milk goal (*n*=235)				
OHM frequency:				
Quartile 2	16.09	12.19	(-7.05, 39.23)	0.187
Quartile 3	22.46	11.42	(-0.57, 45.49)	**0.049**
Quartile 4	29.69	12.94	(3.95, 55.44)	**0.022**
**b. Outcome: Odds of any breastfeeding at discharge**				
	OR	95% CI	*p* value	
Model 1: ≤ 6 months old at discharge (*n*=256)				
OHM frequency:				
Quartile 2	4.77	(1.79, 13.79)	**0.003**	
Quartile 3	6.63	(2.50, 19.27)	**<0.001**	
Quartile 4	6.03	(2.04, 19.30)	**0.002**	
Model 2: ≤ 6 months old + no preoperative enteral feeding (*n*=98)				
OHM frequency:				
Quartile 2	11.38	(2.26, 75.96)	**0.006**	
Quartile 3	9.08	(1.86, 58.76)	**0.011**	
Quartile 4	11.07	(1.88, 83.95)	**0.012**	
Model 3: ≤ 6 months old + any breastfeeding goal (*n*=218)				
OHM frequency:				
Quartile 2	3.35	(1.12, 10.89)	**0.035**	
Quartile 3	5.42	(1.78, 18.16)	**0.004**	
Quartile 4	5.65	(1.65, 20.98)	**0.007**	

a. Analysis was linear regression with robust standard errors for outcome a, and logistic regression for outcome b. All Models were adjusted for neonatal surgical length of stay, insurance type, and race (BIPOC = Asian, Black/African American, Another race or Multirace; White). Models 1 and 3 were also adjusted for any direct breastfeeding during the first 7 days. Models for human milk mL/kg/d were adjusted for age at surgery, and models for breastfeeding at discharge were adjusted for a diagnosis of vocal cord dysfunction.

b. Human milk volumes at discharge are defined as the average mL/kg/d of human milk during the week before discharge. Breastfeeding at discharge is defined as any breastfeeding during the week before discharge.

c. The reference group for OHM frequency is Quartile 1 (0–1 doses). Quartile 2 received 2–6 doses, Quartile 3 received 7–13 doses, and Quartile 4 received ≥14 doses.

*CI* confidence interval, *OHM* oral care with human milk, *OR* odds ratio, *SE* standard error.

Bold values indicate *p* < 0.05.

OHM frequency was also associated with direct BF at discharge (Table 2b). In the full sample, infants in Q2–4 had an estimated 4.77–6.03 greater odds of any BF at discharge (*p* < 0.01 for all quartiles). The magnitude of effect was highest for infants with no preoperative feeding (9.08–11.07 greater odds, *p* < 0.05 for all quartiles). Findings remained consistent when the sample was limited to infants with a documented maternal BF goal (3.35–5.65 greater odds, *p* < 0.05 for all quartiles).

## Discussion

In this study, we characterized the prevalence and patterns of OHM in 297 infants undergoing cardiac surgery over a 10-year period. Although nearly all patients in this cohort received at least 1 OHM dose during the hospitalization, OHM comprised only 25.5% of all oral care during the first 6 postnatal months. Oral care substances other than human milk were considerably more common, particularly after the first postnatal week. This low prevalence of OHM likely reflects the fact that OHM is not a standard pediatric CVICU practice, in contrast to the NICU. To our knowledge, there is only one relevant CCHD study, in which OHM was incorporated into a human milk-focused preoperative feeding protocol [23]. The authors reported that the proportion of infants receiving OHM increased from 0% to 87.3% post-protocol and that OHM was associated with 8.54 shorter days to full feeding volume. Four additional publications from an author group in China investigated OHM compared to oral care with saline for infants with CCHD; however, striking overlap among these manuscripts have led to one paper retraction and an expression of concern about the validity of these publications. Thus, OHM practices across CVICUs are largely undescribed, and the optimal OHM dose or duration for infants with CCHD is unknown.

We found that more frequent OHM in the first 7 postnatal days was associated with significantly higher human milk volume and increased odds of direct BF at hospital discharge. The approximately 20–60 mL/kg/d increase in human milk in our models represents up to one-third of an infant’s total daily nutrition, which is a substantial volume that would otherwise be displaced by commercial formula. While a portion of this volume was estimated due to unmeasured BF sessions, only 38% of infants were BF at discharge, with a median of around 1 session per day. Therefore, the estimated BF volume represents a relatively small proportion of the total nutrition volume. Importantly, excluding estimated BF volumes from the models did not alter the primary findings. Associations also remained consistent when analyses were restricted to infants with a documented maternal human milk or BF goal, suggesting that the findings were not driven entirely by maternal intent or human milk availability. The results of this study underscore the foundational importance of early life experiences for critically ill infants, highlighting the necessity of intentionality in early feeding and developmental care [38], particularly as feeding and nutrition can be challenging for critically ill infants during neonatal hospitalization and throughout childhood [39].

Our findings align with two prior observational studies. Following the implementation of a 5-day OHM protocol for very low birth weight infants, Synder et al. [21] reported increased rates of majority ( > 50%) human milk feeding at 6 weeks (67% vs. 55%, *p* = 0.03) and at discharge (53% vs. 32%, *p* = 0.007), compared to a historical cohort. Similarly, in a large, multisite cohort of preterm and sick term infants (*n* = 482; including 3 with cardiac anomalies), Heine et al. [22] found that early colostrum provision was associated with significantly higher rates of exclusive direct BF at discharge (38.4% vs. 10.7%, *p* = 0.002) although results were limited by lack of covariate adjustment, missing data, and absence of formal OHM procedures. Finally, a small qualitative study (*n* = 30) of parents who successfully breastfed an infant with CCHD reported that 63% of infants received OHM during the hospitalization [40], further supporting a potential link between OHM and direct BF outcomes.

Several potential mechanisms may explain the association between OHM and increased human milk volume. Parental factors likely play a key role, and the relationship between OHM and human milk may be partially bidirectional. Parents who are motivated, well supported by the healthcare team, and physically able to produce human milk may be more likely to provide early human milk/colostrum for OHM, thus increasing their infant’s opportunity for OHM. As early and frequent milk expression is associated with improved milk supply for preterm infants [41–43], the act of expressing milk for OHM could increase human milk volume. Furthermore, providing OHM has been shown to be meaningful for parents [44, 45], with Froh et al. [45] finding that OHM was a strong facilitator of continued human milk expression for mothers of infants with congenital diaphragmatic hernia. Thus, providing OHM may create a positive feedback loop that contributes to successful lactation, improved milk supply, and potential for later BF. This represents an opportunity for intervention, with healthcare team support of parent engagement in OHM potentially facilitating human milk and BF duration in this vulnerable population.

In addition to parental factors, we hypothesize that early, positive oral experiences could contribute to improved oral feeding outcomes for infants with CCHD, including direct BF. The theory of feeding imprinting [46, 47] suggests that an infant’s earliest exposures to feeding stimuli may influence long-term feeding behaviors and preferences. Infants are biologically inclined toward human milk appropriate for their developmental stage, with studies demonstrating newborns’ preferences for the odor of colostrum over mature milk or formula, and for milk from their own mother compared to human milk from another source [48, 49]. Furthermore, a 2022 meta-analysis found that exposure to the smell and taste of human milk reduces pain and improves physiological stability (eg, heart rate, oxygen saturation) for hospitalized newborns [50]. It is plausible that early exposure to human milk via OHM could help counteract potentially aversive oral experiences commonly encountered in intensive care settings, and this is an intriguing question for future research.

Finally, OHM has been associated with improvements in feeding tolerance/timing and gut microbiome composition in preterm infants [5–7, 9–11, 51]. These findings raise the possibility that increased OHM frequency could similarly promote feeding tolerance in CCHD, potentially reducing the need for nutritional adjustments such as transition to elemental formulas. Future research is needed to evaluate the impact of standardizing early and frequent OHM dosing for all infants with CCHD during any periods when they are unable to feed orally, including while receiving enteral feeding on clinical outcomes, such as feeding intolerance. Such studies may be challenging, however, due to diagnostic and clinical heterogeneity resulting in variability in NPO timing and duration, and relatively low incidence of many relevant outcomes such as NEC or sepsis.

### Limitations

This study represents the first known effort to describe patterns of OHM in infants with CCHD. The analysis includes 10 years of detailed, daily human milk and BF data, offering a granular view of early nutrition in this high-risk population. However, several limitations should be considered. Social determinants of health, which are known to influence lactation outcomes, may have contributed to differences in prevalence of both OHM and human milk/BF. For example, in Q1 White infants were underrepresented and infants with public insurance were overrepresented, compared to the overall cohort. While our models were adjusted for these covariates, unmeasured factors such as language barriers, maternal education, transportation challenges, and employment constraints may also play a role. We did not have information on maternal comorbidities known to negatively affect lactation. Breastfeeding volumes were estimated based on previous evidence, which introduces some uncertainty into the results; however, our sensitivity analysis demonstrates that this uncertainty is likely negligible.

Site OHM practices and lactation support changed over time. For example, a “Feeding Champion’s Class” was initiated in 2021 for all new nursing staff. Nurses were instructed to dip a cotton swab or pacifier in maternal human milk, with gentle provision of OHM following the infant’s cues to support positive infant oral experiences and facilitate family engagement. This Feeding Champion’s Class built upon informal, ongoing education about OHM from the unit’s primary feeding therapist, who was consistently working in the unit throughout the study period. In 2023, at the end of the study period, there was a nurse-led project to formally protocolize OHM, which included electronic nursing education with recommendations to provide maternal HM via syringe (0.1 mL into each buccal cavity) every 4 h until age 28 days or until oral feeding initiation. Despite this increasing cultural support, OHM was never officially protocolized, and adjusting for infant birth year did not change or improve the models. The maximum possible OHM doses varied among infants, and it is impossible to know whether infants in a lower quartile had missed opportunities for OHM, or were largely feeding orally and not eligible for OHM. This was not a causal analysis, and the relationship between OHM and human milk volume may be multidirectional. Although the sample size was relatively large for a rare disease population, this study may not generalize beyond this single site. Finally, EHR data is subject to measurement error, varied documentation among clinicians, and practice change over time.

## Conclusion

In this 10-year, single-site study of infants undergoing neonatal surgery for CCHD, increased OHM frequency during the first 7 postnatal days was strongly associated with greater human milk volume and higher BF rates at hospital discharge. These associations persisted even when analyses were limited to infants with a documented maternal goal to provide human milk or BF. Our findings suggest that OHM has potential to support human milk/BF exposure and duration for infants with CCHD. However, OHM was used in only approximately 25% of oral care occurrences with notable differences related to infant race and insurance type. These findings highlight an opportunity for practice change, along with a need for investigation into barriers to and facilitators of OHM. Future research and quality improvement should explore the impact of OHM on clinical outcomes and oral feeding development for vulnerable infants with CCHD.

## Data Availability

The code used for statistical analysis can be accessed via reasonable request from the corresponding author. Analyses were completed in R version 4.5.0.

## Data availability

Due to patient privacy regulations and institutional data-use restrictions, the data for this study cannot be made publicly available and cannot be shared.
